# Physiological and Proteomic Insights Into Red and Blue Light-Mediated Enhancement of *in vitro* Growth in *Scrophularia kakudensis*—A Potential Medicinal Plant

**DOI:** 10.3389/fpls.2020.607007

**Published:** 2021-01-20

**Authors:** Abinaya Manivannan, Prabhakaran Soundararajan, Yoo Gyeong Park, Byoung Ryong Jeong

**Affiliations:** ^1^Institute of Agriculture and Life Science, Gyeongsang National University, Jinju, South Korea; ^2^Division of Applied Life Science (BK21 Plus), Graduate School, Gyeongsang National University, Jinju, South Korea; ^3^Research Institute of Life Science, Gyeongsang National University, Jinju, South Korea

**Keywords:** antioxidant enzymes, trichomes, protein, phenols, flavonoids

## Abstract

The current study has determined the effect of red and blue lights on the enhancement of growth, antioxidant property, phytochemical contents, and expression of proteins in *Scrophularia kakudensis*. *In vitro*-grown shoot tip explants of *S. kakudensis* were cultured on the plant growth regulator-free Murashige and Skoog (MS) medium and cultured under the conventional cool white fluorescent lamp (control), blue light-emitting diodes (LED) light, or red LED light. After 4 weeks, growth, stomatal ultrastructure, total phenols and flavonoids, activities of antioxidant enzymes, and protein expressions were determined. Interestingly, blue or red LED treatment increased the shoot length, shoot diameter, root length, and biomass on comparison with the control. In addition, the LED treatments enhanced the contents of phytochemicals in the extracts. The red LED treatment significantly elicited the accumulation of flavonoids in comparison with the control. In accordance with the secondary metabolites, the LED treatments modulated the activities of antioxidant enzymes. Moreover, the proteomic insights using two-dimensional gel electrophoresis system revealed the proteins involved in transcription and translation, carbohydrate mechanism, post-translational modification, and stress responses. Taken together, the incorporation of blue or red LED during *in vitro* propagation of *S. kakudensis* can be a beneficial way to increase the plant quality and medicinal values of *S. kakudensis*.

## Introduction

*Scrophularia kakudensis* (scrophulariaceae) is a pharmaceutically important plant with several vital secondary metabolites. The extracts of *S. kakudensis* have been widely used to treat ailments such as inflammation, fever, and gastro-intestinal problems (Manivannan et al., 2015b). *S. kakudensis* consists of pharmaceutically important compounds such as acacetin and scrophulasaponins. Besides its pharmaceutical importance, *S. kakudensis* is an unexplored medicinal plant due to inadequate healthy plant materials. Hence, improvement of the micropropagation of *S. kakudensis* by implementation of novel strategies such as the application of different light qualities during propagation could enhance the quality of plant materials. Light is the principal factor for photosynthesis and photomorphogenesis. It also entrains a cascade of reactions involved in primary and secondary metabolism, respectively, in plants. Moreover, the light signals can influence the growth and development of plants in different forms such as quality, quantity, photoperiod, and direction.

Even though several forms of light exist, in recent days, the light quality-mediated influence of physiological, biochemical, and metabolic processes has gained prime importance among plant biologists. Particularly, under an *in vitro* environment, light triggers different metabolic activities of the plant (Kozai et al., 1997). Several reports have shown an improvement of *in vitro* growth in diverse plants by various combinations of light spectra (Batista et al., 2018; Ali et al., 2019; Chen et al., 2020; Silva et al., 2020). In addition, light quality can be applied for the elicitation of vital phytochemicals with pharmaceutical importance. Recently, the application of light quality for the enhancement of secondary metabolites with medicinal importance in a controlled environment such as plant factories, green houses, and *in vitro* culture environment is increasing (Im Chung et al., 2020; Park et al., 2020; Pia̧tczak et al., 2020; Tohidi et al., 2020). Red and blue wavelengths (650 and 450 nm, respectively) are often associated with the enhancement of physiological processes in plants (Kim et al., 2004). Moreover, plants grown under different light qualities in *in vitro* environment consisted of enhanced growth, secondary metabolites, and active antioxidant enzyme metabolism (Kozai et al., 1997; Mengxi et al., 2011; Manivannan et al., 2015a). Recently, the usage of light-emitting diodes (LEDs) over the conventional cool white fluorescent light source has been increasing due to its advantages, such as less heat radiation, energy efficiency, monochromatic spectrum, and longer life span, thus offering a wide range of applications for the plant growers (Kim et al., 2004; Samuoliene et al., 2012; Batista et al., 2018). The application of different light qualities separately or in combination influences the physiology and metabolite contents. For instance, the growth of plants cultivated under red light increased the biomass in lettuce and radish (Goins et al., 2001) and the contents of secondary metabolites in *Ajuga bracteosa* (Ali et al., 2019). Polyphenolic compounds present in plants are associated with diverse functions in growth, defense, protection from UV radiation, and reproduction (Cheynier et al., 2013; Sharma et al., 2019; Tanase et al., 2019). Moreover, the light quality-mediated stimulation of the antioxidant system resulting in the production of secondary metabolites has been shown in several plants (Chen et al., 2020; Silva et al., 2020). In green vegetables, the supplementation of blue light increased the biomass and enhanced the content of vitamin C (Li and Kubota, 2009). Similarly, the green and red LEDs enhanced the contents of sesquiterpenes and monoterpene sabinene in *in vitro* cultures of *Achillea millefolium* (Alvarenga et al., 2015). The red light elicited the levels of carvacrol content in *Plectranthus amboinicus* (Silva et al., 2017), whereas the application of blue light significantly increased the carvacrol content in *Lippia gracilis* (Lazzarini et al., 2018). The blue light enhanced the myrcene and limonene contents in *in vitro*-grown *Lippia rotundifolia*, whereas the combination of red and blue lights in 1:2.5 ratio increased the z-ocimenone metabolite content (de Hsie et al., 2019).

Although several reports have suggested the light quality-mediated modulation of growth, phytochemicals, and reactive oxygen species (ROS) metabolism, very few studies on the proteomics aspects of light quality in *in vitro*-cultured plants are available. For instance, the application of red and blue LEDs improved the maturation and conversion of somatic embryos in sugarcane (Heringer et al., 2017). According to Heringer et al. (2017), the shotgun proteomics investigation of somatic embryos in different stages revealed the up-regulation of proteins involved in the differentiation and de-differentiation process, respectively, in sugarcane. A recent comparative proteomics report by Mirzahosseini et al. (2020) suggested that the supplementation of red and blue light enhanced the wound healing response in *in vitro*-grown *Arabidopsis thaliana* by the improvement of light quality-based defense strategies such as jasmonate-independent signaling pathway. In general, proteins are the functional products of the gene which act as enzymes, primarily for various metabolisms in living organisms. In the present era of modern technologies, application of proteomics has been utilized for a better understanding of the molecular mechanism behind various biological processes in plant biology. Therefore, in the present study, red and blue light-mediated changes in physiology, phytochemicals, and protein expression were studied in the *in vitro*-grown *S. kakudensis.*

## Materials and Methods

### Plant Materials and Light Treatments

The nodal explants obtained from *in vitro S. kakudensis* were sub-cultured onto the plant growth regulator-free Murashige and Skoog (MS) (Murashige and Skoog, 1962) basal medium with 3% (w/v) sucrose and 0.8% (w/v) agar and placed under either cool white fluorescent light (FL) (40-W tubes, Philips, Netherlands) or monochromatic spectral light-emitting diodes (LEDs; PSLED-1203-50A, Force Lighting Co., Ltd., Hwaseong, South Korea), such as red (621–710 nm) or blue (450–475 nm), with the light intensity of 50 μmol m^–2^ s^–1^ PPFD. The light treatments were set up in a completely randomized design with five containers per treatment containing five explants in each container. The distance between the light source and the culture container was 30 cm. Furthermore, the cultures were maintained in a single plant growth chamber with different light quality installed in separate shelves. All the cultures were maintained at 25°C under a 16-h photoperiod with 80% relative humidity. The plants were harvested after 4 weeks for physiological, biochemical, phytochemical, and proteomics analyses.

### Scanning Electron Microscopy Observation

For SEM analysis, the leaf samples were fixed in 2.5% glutaraldehyde overnight at 4°C and washed with 0.1 M phosphate-buffered saline (pH 7.0), followed by staining in 4% osmium tetroxide solution for 2 h at 4°C, and the stained samples were dehydrated in a graded series of ethanol. After dehydration, the samples were dried, gold coated, examined, and photographed under a scanning electron microscope (JSM-6380, JEOL, Tokyo, Japan) operating at 15–25 kV according to Mujib et al. (2014).

### Determination of Antioxidant Enzyme Activities

The extraction of antioxidant enzymes such as superoxide dismutase (SOD), catalase (CAT), ascorbate peroxidase (APX), and phenylalanine lyase (PAL) was performed according to Manivannan et al. (2015a). The total protein content of the samples was estimated by Bradford’s method (Bradford, 1976). The SOD activity was assayed by following the protocol of Giannopolitis and Ries (1977) with the nitro blue tetrazolium inhibition method. The activity of GPX was estimated based on the amount of enzyme required for the formation of tetraguaiacol per minute according to Shah et al. (2001). The activity of CAT was estimated according to the method of Cakmak and Marschner (1992). The activity of APX was estimated by following the protocol of Nakan and Asada (1981). The PAL enzyme activity was determined according to Zhan et al. (2012).

### Estimation of Total Phenols and Flavonoids

The leaf samples were extracted with methanol according to Manivannan et al. (2015b), and the total phenol content of the extract was estimated by the Folin–Ciocalteu principle according to Kumaran and Karunakaran (2007). Furthermore, the total flavonoid content was determined by the aluminum chloride method mentioned by Manivannan et al. (2015a). The total phenol and flavonoids were determined from a standard gibberellic acid and quercetin calibration curve, respectively.

### Proteomics

#### Protein Extraction

For 2D-PAGE analysis, protein extraction was carried out by following the procedure of Muneer et al. (2015). In detail, the leaf tissue (0.1 g) was homogenized in liquid nitrogen using a pre-chilled pestle and mortar. The proteins were extracted with a commercial protein extraction kit (ReadyPrep, Bio-Rad, Hercules, CA, United States) according to the instructions provided by the manufacturer. For total protein isolation, about 2 ml of extraction buffer {8 M urea, 4% 3-[(3-cholamidopropyl) dimethylammonio]-1-propanesulfonate (CHAPS), 40 mM Tris, 0.2% bio-lyte (*pI* 3-10)} was mixed with the lyophilized (0.1 g) leaf tissue. The homogenate was vortexed and sonicated with an ultrasonic probe to disrupt any interfering substances such as genomic DNA and phenolics. After sonication, the samples were centrifuged for 30 min at 4°C, and the supernatant was transferred to new Eppendorf tubes. The resultant supernatant was employed for isoelectric focusing after protein quantification with the Bradford method using a bovine serum albumin standard curve.

#### Isoelectric Focusing and Two-Dimensional Gel Electrophoresis

A total of 70 μg of dissolved protein sample was separated by the 2-DE in the first dimension by the isoelectric focusing on a 7-cm IPG strip (*pI* 4–7) (GE Healthcare, Little Chalfont, Buckinghamshire, United Kingdom) and the second dimension by SDS-PAGE on a Protean II unit (Bio-Rad, Hercules, CA, United States), according to methods given by Muneer et al. (2015). The samples were rehydrated for 12 h (with a 125-μl rehydration buffer containing 70 μg proteins) before focusing. For the first dimension, the rehydrated strips were focused at 20°C with 50 μA current per strip using a four-step program: step and hold—300 V for 30 min, gradient—1,000 V for 30 min, gradient—5,000 V for 1 h 30 min, and final step and hold for 1–2 h until the final voltage reached 10,000 V. The focused strips were equilibrated twice for 15 min in 10 mg ml^–1^ DTT and then in 40 mg ml^–1^ iodoacetamide prepared in an equilibration buffer containing 50 mM Tris-HCl (pH 8.8), 6 M urea, 30% (v/v) glycerol, and 2% (w/v) SDS. After equilibration, the strips were attached to the second dimension gel (12.5%) with a 0.5% low melting point agarose sealing solution. Electrophoresis was done at a constant voltage of 80 V for 4 h until the bromophenol dye front reached the end of the gel. The protein spots in the analytical gels were stained using the silver staining method.

### Image Acquisition and Data Analysis

Three replicate gels from each treatment were used for image acquisition and data analysis. Spot detection, spot measurement, background subtraction, and spot matching were performed using Progenesis SameSpotsQI 2D software (ver. 4.1, Nonlinear Dynamics, Newcastle, United Kingdom) in an automatic spot detection mode to review the annotations of spots statistically using one-way analysis of variance (ANOVA) analysis (*n* = 3, *p* < 0.05) at 95% confidence level. The differentially expressed protein spots were identified as spots showing more than a twofold change in expression in comparison with the control.

### In-Gel Digestion and MALDI-TOF-MS Analysis

The differentially expressed protein spots were excised manually from the gels and washed with distilled water three times. The protein spots were chopped and de-stained with 30 mM potassium ferricyanide and 100 mM sodium thiosulphate pentahydrate (1:1) by incubating at room temperature for 30 min. The de-staining reagent was removed, and the gel particles were treated with 100 μl of 50 mM NH_4_HCO_3_ for 5 min and dehydrated in 30 μl of acetonitrile for 5 min. After dehydration, the gel was covered with a 100-μl reduction solution (10 mM dithiothreitol in 50 mM NH_4_HCO_3_) and incubated for 45 min at 56°C. After the removal of the reduction solution, 100 μl of alkylation solution (100 mM iodoacetamide in 50 mM NH_4_HCO_3_) was added and incubated at 25°C in the dark for 30 min. Finally, the gel pieces were washed with 30 μl of 50 mM NH_4_HCO_3_ for 5 min and dehydrated with 30 μl of acetonitrile for 10 min. After drying using a vacuum centrifuge, the gel pieces were rehydrated in 5–10 μl of 25 mM NH_4_HCO_3_ containing 5 ng μl^–1^ trypsin (Promega, Madison, WI, United States) at 37°C for 30 min. After incubation, the excess trypsin solution was replaced with 5–10 μl of 25 mM NH_4_HCO_3_, and digestion was carried out for a minimum of 16 h at 37°C. The digested peptides were subsequently pooled, vacuum dried, and mixed with 3 μl of sample solution (50% acetonitrile and 0.1% trifluoroacetic acid). For protein identification, the tryptic-digested peptide mixtures were targeted onto a matrix-assisted laser desorption/ionization–time-of-flight mass spectrometry (MALDI-TOF-MS) plate (AccuSpot, Shimadzu Ltd., Kyoto, Japan) and analyzed by a Voyager-DE STR mass spectrometer (Applied Biosystems, Franklin Lakes, NJ, United States) equipped with delay ion extraction.

### Peptide Identification and Gene Ontology Analysis

Mass spectra were obtained over a mass range of 800–3,500 Da. Homology search was executed by matching the experimental results with both theoretical digests and sequence information from the public protein databases using the Mascot software^1^. The search parameters employed were as follows: carbamidomethyl cysteine as a fixed modification and oxidation of methionine as a variable modification, one missed cleavage site, and peptide mass tolerance of ±100 ppm. The NCBI-nr database^2^ with the taxonomy Viridiplantae was employed to identify regions of similarity between sequences. The protein score employed was −10^∗^log(*P*), where *P* is the probability that the observed match is a random event. The spot identities were submitted to an agbase gene ontology (GO) retriever, and the resulting annotations were summarized based on the GOSlim set using GOSlim Viewer (Muneer et al., 2015).

### Statistical Analysis

All the assays were performed in triplicate, and the results were averaged. Significant differences among the treatments were determined by ANOVA, followed by Duncan’s multiple-range test, at a significance level of 0.05 using the Statistical Analysis System computer package (V.6.12, SAS Institute Inc., Cary, NC, United States).

## Results and Discussion

### Influence of Light Qualities on Growth and Morphology

**FIGURE 1:**
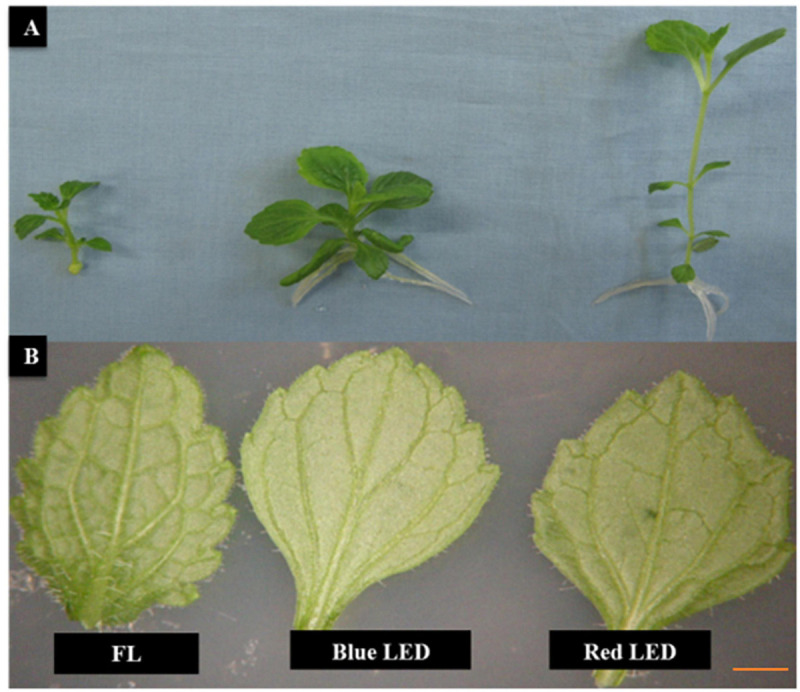
Effect of light quality on the growth of *Scrophularia kakudensis*. Morphological differences observed in *S. kakudensis* plantlets **(A)** and variation in the leaf morphology **(B)** of *S. kakudensis* as affected by different light qualities.

The light qualities influenced the growth parameters and morphology of *S. kakudensis* (Table 1 and Figure 1A). Among the light treatments, the red LED treatment produced slender plants with longer stems. The red LED treatment increased the shoot length by 82.0 and 100% than the blue LED and FL treatment, respectively. Similarly, the red LED increased the stem length in *Rehmannia glutinosa*, a medicinal plant from the same Scrophulariaceae family (Manivannan et al., 2015a). Previous reports demonstrated that red light induced the levels of plant growth regulators, particularly endogenous gibberellins, which play a vital role in cell elongation by stimulating the mitosis in both apical and sub-apical meristem, which could result in taller stems (Arney and Mitchell, 1969; Toyomasu et al., 1993). On the other hand, the blue LED treatment resulted in shorter shoots with a significantly larger diameter in comparison with the other treatments. Supplementation of blue LED increased the shoot diameter by 11.5% than the FL and by 109.6% in comparison with the red LED treatment, whereas the FL treatment increased by 88.0% than the red LED treatment. Similar effects of blue light were recorded in *R. glutinosa* (Manivannan et al., 2015a), and blue light-mediated increase in biomass was reported in Chinese cabbage (Li et al., 2012). Moreover, LED treatments induced early rooting in *S. kakudensis*. Red LED enhanced the number and length of roots by 40.0 and 50.0% in comparison with the blue light, whereas roots were not observed in the control plants. The stimulation of roots in the LED treatments could be due to the involvement of photoreceptors, auxin signaling, and light piping mechanism in which the transmission of light signals occurs through the internal tissues from the aboveground shoot to the root in a wavelength-dependent manner; particularly the long-wavelength red light and far red are well transmitted (Sun et al., 2005; Canamero et al., 2006; van Gelderen et al., 2018). Similar observations were reported in *Triticum aestivum* upon blue light irradiation (Dong et al., 2014). Furthermore, the biomass of *S. kakudensis* was significantly increased by the blue LED treatment followed by the red light. In detail, the blue LED treatment enhanced the fresh weight by 91.2 and 95% in comparison with the FL and red LED treatments. Similarly, the dry weight was increased by 72.0 and 87.0% than the FL and red LED treatment, respectively. Concordantly, Wheeler et al. (1991) also suggested the effectiveness of the blue and red light sources for healthy plant growth. The improvement of fresh and dry weights by the application of blue or red LED treatment alone or in combination was reported in *Oncidium* (Mengxi et al., 2011). Overall, the use of blue or red LED during *in vitro* propagation can be beneficial for the improvement of plant production. The application of blue and red light for the *in vitro* growth of medicinal plants has been reported in *Ajuga bracteosa* (Ali et al., 2019), *Pfaffia glomerata* (Silva et al., 2020), *Fritillaria cirrhosa* (Chen et al., 2020), and *Lippia rotundifolia* (de Hsie et al., 2019).

**TABLE 1:** Growth traits measured after 4 weeks of light treatment in *Scrophularia kakudensis*.

**Light treatment**	**Shoot length (cm)**	**Stem diameter (mm)**	**Length of the longest root (cm)**	**Number Of roots**	**Total fresh weight (g)**	**Total dry weight (mg)**
FL	2.0 ± 0.03^*a*^b^*b*^	0.156 ± 0.07b	0.0 ± 0.00c	0.0 ± 0.00c	0.102 ± 0.01b	10.0 ± 0.04b
Blue LED	2.2 ± 0.05b	0.174 ± 0.02a	1.2 ± 0.01b	5.0 ± 0.07b	0.195 ± 0.04a	17.2 ± 0.02a
Red LED	4.0 ± 0.06a	0.083 ± 0.05c	1.8 ± 0.03a	7.0 ± 0.05a	0.100 ± 0.05b	9.2 ± 0.04b

**FL, fluorescent lamp; LED, light-emitting diodes. ^*a*^Values represent the mean ± SE of three replications. ^*b*^Means followed by different letter(s) in one measurement indicate statistically significant difference at P ≤ 0.05 by Duncan’s multiple-range test*.*

**FIGURE 2:**
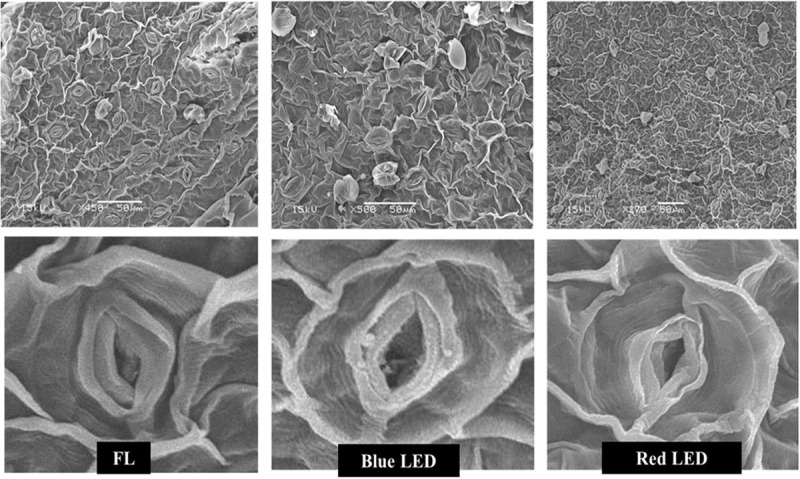
Effect of light quality on stomatal density and structure of *in vitro*-grown *S. kakudensis*.

**FIGURE 3:**
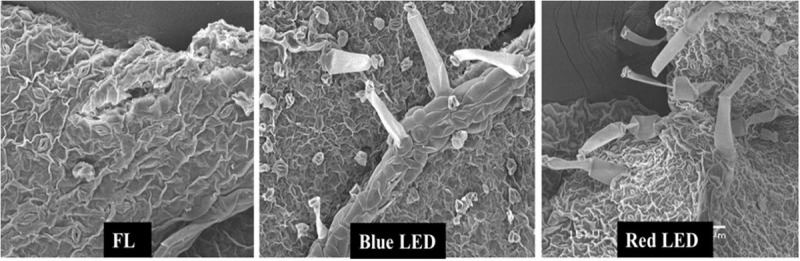
Scanning electron micrographs of the trichomes of *in vitro*-grown *S. kakudensis* as affected by light qualities.

The leaf morphology of *S. kakudensis* was also modulated by the variation in light quality (Figure 1B). Broader leaves were observed in the blue and red light treatments in comparison with the control FL. In general, leaves perceive the light signals from the external environment by means of specialized receptors called photoreceptors, and thus modulation in the light quality readily affects the leaf characteristics (Kim et al., 2004; Park et al., 2012). A larger leaf area increases the absorption of light, which directly influences photosynthesis (Nishimura et al., 2009). The leaf morphological observations reflected on the density of stomata (Figure 2). The stomatal density was significantly enhanced in the blue LED treatment than in the other treatments. Moreover, the scanning electron micrograph of a leaf revealed the occurrence of more numbers of glandular trichomes in the red and blue LED-grown leaves in comparison with the FL (Figure 3). Interestingly, the increase in the density of glandular trichomes by red light was also reported in the *in vitro* cultures of *Alternanthera brasiliana* (Macedo et al., 2011). Moreover, in *Olea europaea*, the density of the trichomes was positively correlated with UV-B irradiation and considered as the defense response exhibited by plants (Liakoura et al., 1997).

### Influence of Light Qualities on Redox Metabolism

**FIGURE 4:**
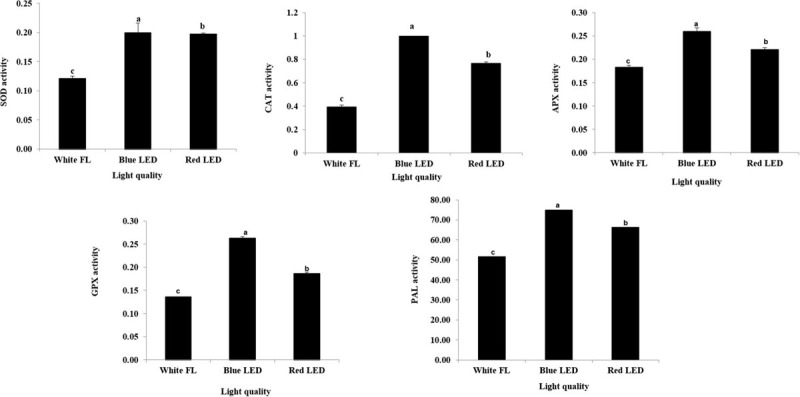
Modulation antioxidant enzyme activities upon different light quality treatments. Data are the mean ± SE from three replicates. Different letter(s) in one measurement indicates statistically significant difference at *P* ≤ 0.05 by Duncan’s multiple-range test.

The blue and the red LED treatments have positively influenced the activities of antioxidant enzymes in *S. kakudensis*. In detail, the highest SOD activity was observed upon the application of blue LED treatment followed by the red LED treatment (Figure 4). Generally, the antioxidant enzymes prevent the cell damages caused by the ROS by catalyzing a cascade of reactions. As the first line of defense, the SOD dismutases the O_2_^–1^ radical into H_2_O_2_ and molecular oxygen (Gill and Tuteja, 2010). Therefore, the higher activity of SOD (H_2_O_2_ generator) in the blue LED treatment followed by the red LED treatment reflects the higher O_2_^–1^ scavenging potential. Subsequently, the generated H_2_O_2_ will be eliminated by the action of GPX and CAT (H_2_O_2_ scavengers) (Shohael et al., 2006). These enzymes were also markedly increased upon irradiation with the blue LED and red LED than the control (Figure 4). Along with the above-mentioned enzymes, APX, an important enzyme involved in the ascorbate–glutathione cycle, also plays a vital role in H_2_O_2_ elimination. In the present study, the APX activity also followed the same trend as the other antioxidant enzymes. Similarly, the PAL enzyme activity was also increased in the blue and red LED treatments. The PAL is a key enzyme responsible for the synthesis of phenols and flavonoids *via* the phenylpropanoid pathway. Earlier reports suggested that the elevated transcriptional regulation of the PAL upon oxidative stress augments the synthesis of secondary metabolites (Zhan et al., 2012). Similarly, the supplementation of combinations of red and blue lights increased the activities of antioxidant enzymes in the *in vitro* plants of *Carpesium triste* (Zhao et al., 2020) and in the callus cultures of *Cinidium officinale* (Adil et al., 2019).

### Influence of Light Qualities on the Contents of Secondary Metabolites

**FIGURE 5:**
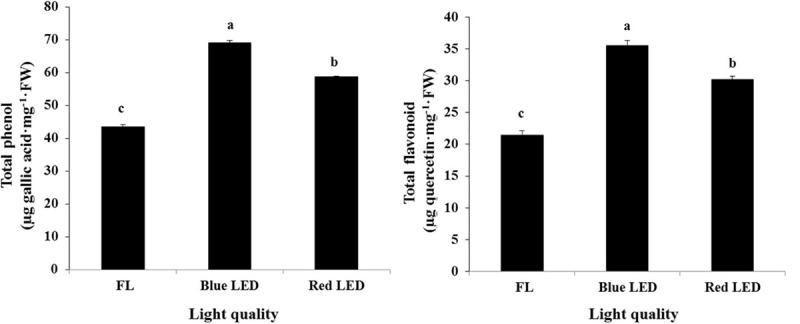
Enhancement of total phenols and flavonoids in *S. kakudensis* upon different light quality treatments. The units of the enzymes are as follows: SOD activity (nmol NBT min^–1^ mg^–1^ protein), CAT activity (nmol H_2_O_2_ min^–1^ mg^–1^ protein), APX activity (nmol ascorbate oxidized min^–1^ mg^–1^ protein), GPX activity (nmol guaical min^–1^ mg^–1^ protein), and PAL activity (μmol cinnamic acid h^–1^ mg^–1^ FW). Data are the mean ± SE from three replicates. Different letters in one measurement indicate statistically significant difference at *P* ≤ 0.05 by Duncan’s multiple-range test.

The light quality-mediated elicitation of secondary metabolites has gained importance in recent days, especially in medicinal plants (Alvarenga et al., 2015; Manivannan et al., 2015a). The light-mediated elicitation of phenols and flavonoids has been reported by Samuoliene et al. (2012) in lettuce. In this experiment, the contents of total phenols and flavonoids were found to be markedly higher in the blue LED treatment, followed by the red LED treatment (Figure 5). Notably, the blue LED immensely increased the total phenols (55.14% higher than the FL) and flavonoids (65.68% higher than the FL). The red LED treatment also resulted in the elicitation of phenols and flavonoids to 31.91 and 40.79%, respectively, than the FL. Ballaré (2014) reported that light signal influences the accumulation of phytochemicals by modulating the phenylpropanoid pathway. In general, majority of the secondary metabolites like phenols are synthesized *via* the phenylpropanoid pathway. Moreover, the accumulation of phenolic compounds by light-induced regulation of the PAL is considered as a vital mechanism behind the phytochemical elicitation (Koyama et al., 2012). This enzyme controls the biosynthesis of polyphenols by catalyzing the flux of primary metabolites into the phenylpropanoid biosynthetic pathway. Furthermore, the transcript levels of the PAL, chalcone synthase, chalcone isomerase, flavone-3-hydroxylase, flavonol synthase, flavonoid-3′-hydroxylase, anthocyanidin synthase, and dihydroflavonol-4 reductase, the key enzymes involved in the biosynthesis of important secondary metabolites, have been significantly influenced by light quality in plants (Quideau et al., 2011; Koyama et al., 2012). In *A. bracteosa*, blue light enhanced the accumulation of total polyphenol contents (Ali et al., 2019). Overall, the results illustrate the indispensable roles of red and blue LEDs over the conventional FL for the enhancement of secondary metabolites in *S. kakudensis*.

### Influence of Light Qualities on Protein Expression

**FIGURE 6:**
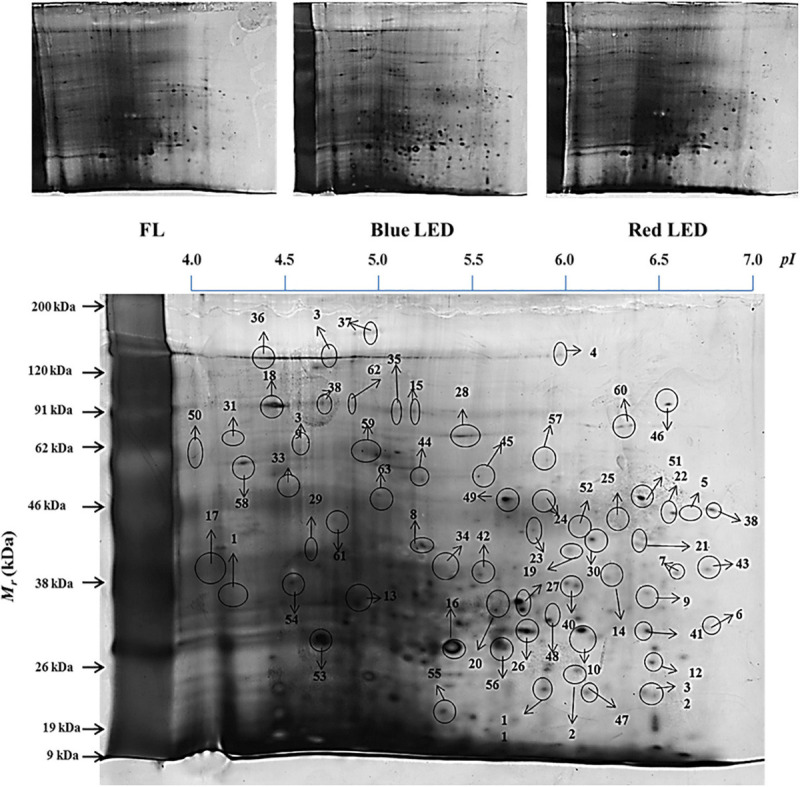
Leaf protein profiles of *S. kakudensis* upon different light quality treatments and the master gel representation (obtained from the blue LED treatment) with identified protein spots.

**FIGURE 7:**
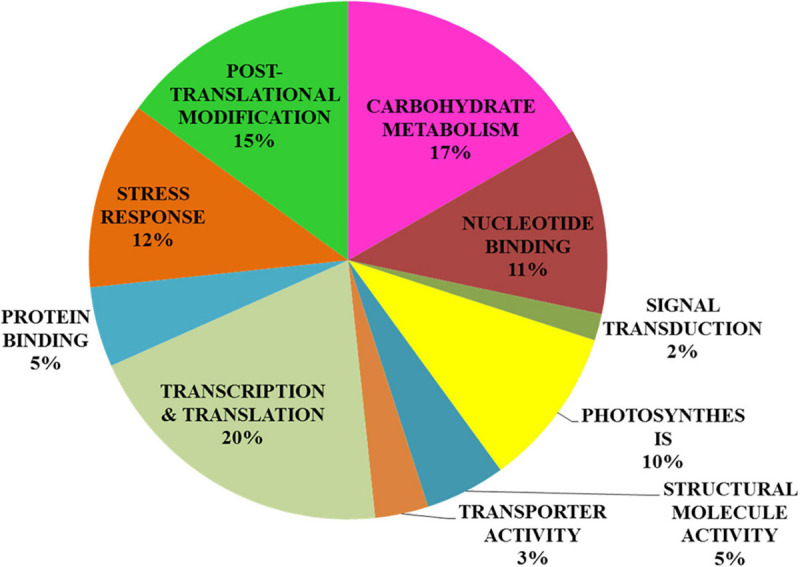
Functional characterization of proteins identified using the matrix-assisted laser desorption/ionization–time-of-flight mass spectrometry analysis from the leaf proteome.

In addition to the above-mentioned physiological and biochemical factors, proteomics tools were utilized to investigate the effect of light quality on *S. kakudensis* leaves (Figure 6). The comparative analysis of 2-DE gels analyzed by Progenesis SameSpots TotalLab (New Castle, United Kingdom) detected about 455 protein spots and was reproducibly resolved among the three replicates. Among the resolved spots, 149 protein spots were differentially expressed with more than 2.0-folds change. From the analyzed 149 spots, proteins from 63 spots were identified using MALDI-TOF-MS (ABI4800, Applied Biosystems, MA, United States). Table 2 shows the list of identified proteins along with the corresponding spot ID, nominal mass, theoretical and calculated *pI*, accession number, MASCOT score, and sequence coverage percentage. The sequence coverage percentages of the identified proteins were in the range of 14–100%. Variation in light quality up-regulated the proteins involved in several metabolic processes, and the putative functional characterization is illustrated in Figure 7. The blue LED and red LED treatments up-regulated the expression levels of 30 and 25 proteins, respectively. However, the FL treatment increased the abundance of 13 protein spots.

**TABLE 2:** Differentially expressed proteins identified using the MALDI-TOF MS from the leaf proteome.

**Spot no.^*a*^**	**Accession number**	**Nominal mass (*M*_*r*_)^*b*^**	**Theoretical *pI*^*c*^**	**Protein identification**	**Species**	**Sequence coverage (%)**	**Mascot score**	**Fold^*d*^**
1	KHN29290	30,653	7.55	ALBINO_3_-like protein 2, chloroplastic	**Glycine soja**	30	68	2.5
2	XP_007510766	32,506	6.86	Nitrogen-fixing NifU domain protein	**Bathycoccus prasinos**	56	44	3.8
3	XP_002532859	25,792	6.11	Proteasome subunit alpha type, putative	**Ricinus communis**	48	50	2.7
4	AAP46638	108,300	8.16	PG1	**Hordeum vulgare**	56	52	5.7
5	XP_007015404	27,608	5.93	20S proteasome alpha subunit G1	**Theobroma cacao**	34	49	2.4
6	AAY62983	23,313	10.24	Small ribosomal protein subunit 4, partial	**Caribaeohypnum polypterum**	48	48	6.5
7	NP_564622	34,819	9.11	RNA-binding S4 domain-containing protein	**Arabidopsis thaliana**	49	36	5.7
8	XP_003058724	31,202	9.40	NADPH-dependent 1-acyldihydroxyacetone phosphate reductase-like	**Erythranthe guttatus**	43	44	2.8
9	XP_012832543	5,912	12.49	Transmembrane protein, putative	**Medicago truncatula**	74	46	6.3
10	ACJ74207	7,631	8.04	LEA-like protein	**Solanum tuberosum**	63	35	2.1
11	EMS51364	3,387	11.01	RNA polymerase II second largest subunit	**Rhododendron viscistylum**	100	35	4.1
12	XP_011047745	90,784	5.73	Nuclear valosin-containing protein-like isoform X1	**Populus euphratica**	59	57	5.4
13	AAU88175	10,369	9.98	Disease resistance-like protein	**Coffea wightiana**	76	45	5.4
14	KMZ70332	41,940	6.56	Glyoxylate/hydroxypyruvate reductase B	**Zostera marina**	50	43	6.8
15	KMZ55951	106,021	6.18	Villin-3	**Zostera marina**	15	43	3.6
16	XP_008386513	28,563	9.40	tRNA 2′-phosphotransferase 1-like	**Malus domestica**	27	53	4.1
17	XP_010088475	27,514	5.93	Proteasome subunit alpha type-3	**Morus notabilis**	62	48	4.9
18	XP_003602824	23,398	5.54	14-3-3-like protein	**Medicago truncatula**	39	52	2.6
19	EMS62457	53,837	5.53	Aldehyde dehydrogenase family 2 member C4	**Triticum urartu**	21	45	5.1
20	XP_004979712	12,518	8.93	ER-localized cyclophilin, partial	**Triticum urartu**	56	45	4.9
21	NP_564622	34,819	9.11	RNA-binding S4 domain-containing protein	**Arabidopsis thaliana**	30	48	4.8
22	BAF01042	19,490	6.60	ACD-ScHsp26-like protein	**Tamarix hispida**	58	46	2.5
23	XP_007029049	83,906	5.99	P-loop containing nucleoside triphosphate hydrolases superfamily protein isoform 2, partial	**Theobroma cacao**	19	53	4.0
24	ABR25818	5,672	4.60	Phenylalanine ammonia-lyase, partial	**Oryza sativa Indica**	100	40	3.5
25	NP_180077	123,947	8.65	Actin-binding FH2 protein	**Arabidopsis thaliana**	32	51	3.6
26	KHN11740	12,360	6.41	Heat shock 22-kDa protein, mitochondrial	**Glycine soja**	88	51	4.5
27	AAC39472	64,970	6.22	Vacuolar protein sorting homolog	**Arabidopsis thaliana**	14	57	3.8
28	P31843	16,960	6.90	RNA-directed DNA polymerase homolog	**Oenothera berteroana**	44	54	4.4
29	NP_001150821	51,409	8.67	CIPK-like protein 1	**Zea mays**	32	53	5.0
30	XP_010094559	39,903	9.34	Protein kinase APK1B	**Morus notabilis**	36	51	3.0
31	AAS60004	2,930	11.72	Photosystem II subunit H, partial (chloroplast)	**Alstroemeria aurea**	100	40	5.2
32	AKP98463	29,663	9.61	Maturase K, partial (chloroplast)	**Duabanga grandiflora**	57	50	4.3
33	AAS98723	23,263	10.19	Ribosomal protein subunit 4	**Touwiodendron diversifolium**	47	47	5.5
34	AES94384	16,893	7.68	RAB GTPase-like protein B1C	**Medicago truncatula**	59	46	3.3
35	XP_001417143	128,844	5.53	Predicted protein	**Ostreococcus lucimarinus**	32	36	3.2
36	CAB90625	41,311	9.32	Phosphoenolpyruvate carboxylase	**Dendrobium loddigesii**	52	48	4.0
37	XP_010091954	50,674	9.72	Chloroplastic group IIA intron splicing facilitator	**Morus notabilis**	49	56	5.2
38	AFA51060	21,277	5.73	RB protein, partial	**Solanum microdontum**	50	47	4.5
39	XP_004290877	12,296	7.79	Acetyltransferase At1g77540	**Fragaria vesca** subsp.**vesca**	88	45	4.4
40	XP_003598507	43,923	6.87	Patellin, partial	**Medicago truncatula**	22	47	7.9
41	XP_010253868	40,572	9.63	Thylakoid ADP, ATP carrier protein, chloroplastic	**Nelumbo nucifera**	42	54	5.1
42	ABU88982	56,588	9.29	Phospholipid/glycerol acyltransferase	**Helianthus annuus**	39	44	6.9
43	KEH24073	19,979	8.57	F-box protein interaction domain protein	**Medicago truncatula**	49	45	6.1
44	AFC90234	34,139	9.22	Nucleotide-binding site leucine-rich repeat protein	**Rhododendron formosanum**	53	41	6.9
45	AIU48049	43,923	6.87	Structural maintenance of chromosomes protein, partial	**Theobroma cacao**	32	42	7.2
46	AAW69888	22,267	10.19	Small ribosomal protein 4	**Zygodon bartramioides**	59	44	4.3
47	BAH80000	23,730	5.61	Putative retrotransposon protein	**Oryza sativa Indica**	50	52	6.0
48	BAD62243	49,128	11.60	Zinc knuckle domain-like	**Oryza sativa Japonica**	73	47	4.3
49	CCA60911	37,841	6.96	Phosphoenolpyruvate carboxylase, partial	**Chasmanthium latifolium**	28	44	3.4
50	AES70881	21,466	9.20	UTP-glucose-1-phosphate uridylyltransferase	**Medicago truncatula**	52	57	5.3
51	KHG14889	47,709	8.77	Advillin	**Gossypium arboreum**	45	49	4.4
52	XP_010111987	69,444	5.50	Exocyst complex component 7	**Morus notabilis**	53	56	2.2
53	CAB86074	53,160	5.65	Importin alpha-like protein	**Arabidopsis thaliana**	26	46	5.7
54	NP_567284	12,053	9.51	Putative copper transport protein	**Arabidopsis thaliana**	58	42	4.4
55	XP_003598507	50,425	9.22	DNA methyltransferase 1-associated protein,	**Ricinus communis**	50	42	4.1
56	XP_002955677	20,823	9.46	Ribulose-1,5-bisphosphate carboxylase/oxygenase	**Volvox carteri**	33	43	6.2
57	KEH35435	18,308	9.46	Proline-rich cell wall-like protein	**Medicago truncatula**	42	52	5.3
58	P09003	22,878	9.53	ATP synthase protein MI25	**Nicotiana tabacum**	58	48	5.7
59	AGC78858	22,441	10.22	Mitochondrial ATPase F(0) complex, subunit 4	**Vicia faba**	63	43	3.9
60	ACP41917	10,933	8.79	RNA polymerase IV, partial	**Heliosperma insulare**	64	37	4.1
61	XP_002311057	23,881	8.52	GTP-binding family protein	**Populus trichocarpa**	58	38	4.7
62	XP_003539430	15,545	7.66	Auxin-induced protein 6B-like	**Glycine max**	36	51	4.1
63	XP_010928981	24,551	11.62	Putative glycine-rich cell wall structural protein	**Elaeis guineensis**	69	45	4.7

**^*a*^The spot no. corresponds to the number labeled in the protein gel images. ^*b*^Theoretical molecular mass (M_*r*_) calculated for the peptides using MASCOT peptide mass fingerprint. ^*c*^Isoelectric point of the peptides calculated using MASCOT peptide mass fingerprint. ^*d*^Fold change between the treatments calculated using the Progenesis Samespot 2D software v4.1 (Nonlinear Dynamics, Newcastle, United Kingdom)*.*

Among the treatments, the monochromatic blue light significantly enhanced the expressions of 30 protein spots. The albino-3-like protein (spot 1), which plays a vital role in the insertion of the light-harvesting complex (LHC) proteins into the thylakoid membrane, was up-regulated by blue light. In addition, the protein is essential for the assembly and activity of LHC I and II. The abundance of a nitrogen-fixing NifU domain containing a protein (spot 2) also increased upon blue LED treatment. Chloroplastic NifU-like proteins act as molecular scaffolds in the biosynthesis of iron–sulfur clusters in plants. In *Arabidopsis*, the mutation in NifU protein resulted in the impairment of photosynthetic electron transport and reduction of steady-state levels of photosystem I (Yabe et al., 2004). Thus, the increase in NifU protein levels in the blue LED treatment could have contributed to the enhancement of the photosynthetic mechanism in *S. kakudensis*. In addition, blue light increased the expression levels of proteasome subunits (spots 3, 5, and 17), indicating the occurrence of a stress response. In general, the 20S proteasome is composed of α and β subunits within which the target proteins are degraded to short peptides *via* a threonine protease activity (Belknap and Garbarino, 1996). The up-regulation of proteasome subunits was observed upon several abiotic stress conditions. Moreover, the blue LED treatment increased the levels of the late embryogenesis abundant (LEA) protein (spot 10). LEA was associated with resistance to various forms of stress, in particular, water stress, desiccation stress, and heat stress (Tunnacliffe and Wise, 2007). The up-regulation of RNA polymerase II (spot 11) in the blue light treatment indicated the transcriptional activation of vital genes involved in the photosynthesis process. The enhancement in the level of phenylalanine ammonia lyase (PAL) (spot 24), an important rate-limiting enzyme in the phenylpropanoid pathway responsible for the synthesis of secondary metabolites such as phenols and flavonoids, corresponds with previous reports suggesting the elicitation of the transcripts of PAL upon the blue and red LED treatment (Rahman et al., 2015). The PAL activity tested in this experiment and the phenol contents correlate with the proteomics result. In addition, the increase in the abundance of ACD-ScHSP26 (spot 22) and heat shock protein (spot 26) also illustrates the occurrence of light-mediated stress that caused the stimulation of molecular chaperones to repair the incorrect protein folding. Protein kinase (spot 30) plays an important role in the phosphorylation of proteins. The phosphorylation of proteins can be triggered by photoreceptors, the light-signaling molecules according to Smith (2000).

In addition to the above mentioned proteins, blue light also up-regulated the expression levels of proteins such as the actin-binding FH2 protein, vacuolar protein sorting homolog, CIPK-like protein, patellin, F-box protein interaction domain, nucleotide-binding site leucine-rich repeat protein, structural maintenance of chromosome, advillin, DNA methyl transferase-1-associated protein, and mitochondrial ATPase F(0) complex. Thus, blue light imposed a stressful environment that resulted in the activation of several proteins related to stress tolerance, photosynthesis, gene regulation, post-translational modification, and secondary metabolism in *S. kakudensis*. The blue LED treatment enhanced the accumulation of phosphoenol pyruvate carboxylase (spot 36). Phosphoenol pyruvate carboxylase is a rate-limiting enzyme involved in the carbon fixation process and Crassulacean acid metabolism, a well-known adaptation mechanism in plants, especially under water stress conditions (Cushman et al., 1989). The stimulation of phosphoenol pyruvate was reported as an important strategy adopted by plants to combat the energy expense during stress (Cushman et al., 1989).

The irradiance of red LED increased the expression of small ribosomal protein (spot 6) and disease resistance protein (spot 13) which play vital roles in protein synthesis to overcome the oxidative stress caused by the external environment. Glyoxylate/hydroxyl pyruvate reductase B (spot 14) catalyzes the NADPH-dependent reduction of glyoxylate and hydroxypyruvate into glycolate and glycerate, respectively. It is indispensable for the mediation of the passage of carbons through the carbon oxidation pathway during photorespiration (Givan and Kleczkowski, 1992). In addition, red light enhanced the levels of photosystem II subunit (spot 31). PS II is a vital multi-protein enzyme involved in the photosynthetic process. Thus, the enhancement of PS II protein could improve photosynthesis. Overall, in the red LED treatment, the protein increases were associated primarily with photosynthesis, carbohydrate metabolism, protein repair, and stress resistance.

On the other hand, FL treatment improved the expressions of proteins involved in carbon fixation and photosynthesis, namely, the NADPH-dependent acyldihydroxyacetone phosphate reductase (spot 8) and phosphoenol pyruvate carboxylase (spot 49). Moreover, the GTP-binding family protein (spot 61), small ribosomal protein (spot 46), ER-localized cyclophilin (spot 20), phospholipid/glycerol acyltransferase (spot 42), exocyst complex component (spot 52), RNA polymerase IV (spot 60), auxin-induced protein (spot 62), and putative glycine-rich cell wall structural protein 1 (spot 63) were significantly increased in the control FL treatment. Thus, the regulation of the above-mentioned proteins involved in photosynthesis, post-translational modification, carbohydrate metabolism, development, and stress resistance process provides a molecular insight into the light quality-induced protein expression modulation in *S. kakudensis* leaf.

**FIGURE 8:**
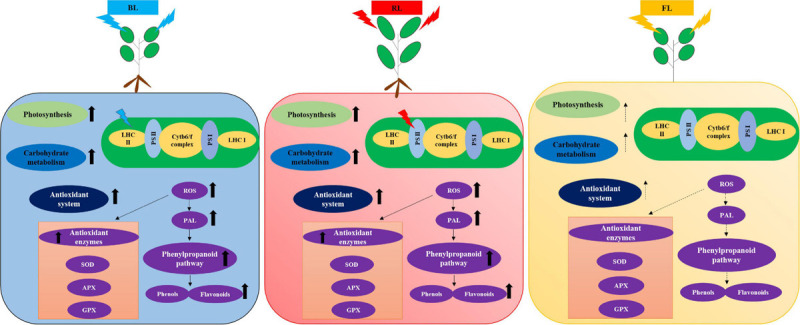
Schematic representation of light quality-mediated effects in the photosynthesis and antioxidant system of *in vitro*-grown *S. kakudensis* hypothesized based on the outcomes of the present study. Blue light-mediated influence on the light harvesting complex II (LHC II) and red light-mediated influence on photosystem II (PS II) on the electron transport chain have been depicted based on the proteomics results. Cytb6/f complex, cytochromeb6/f complex; PS I, photosystem; PS II, photosystem II; LHC II, light-harvesting complex II; LHC I, light-harvesting complex I; SOD, superoxide dismutase; APX, ascorbate peroxidase; GPX, guaiacol peroxidase; ROS, reactive oxygen species; PAL, phenylalanine ammonia lyase.

Taken together, the light quality-mediated photomorphogenesis process could facilitate the *in vitro* culture of medicinal plants like *S. kakudensis*. Moreover, the tailored use of blue and red LED lights can be utilized for the improvement of photosynthesis, photomorphogenesis, gene expression, and regulation of various metabolisms (Batista et al., 2018). The supplementation of different light qualities tends to activate the photoreceptors in plants such as red light-responsive phytochromes and blue light-mediated cryptochromes and phototrophins. These photoreceptors influence the photochemical control of various gene expressions, leading to the regulation of functional proteins in various metabolic pathways involved in the biosynthesis of primary and secondary metabolites (Batista et al., 2018). In addition, the *in vitro* culture environment poses a certain level of stress to the explants which also acts as trigger for the activation of diverse antioxidant systems in plants, leading to the elicitation of secondary metabolites with nutraceutical values. Based on the physiological, biochemical, and proteomic analysis, the application of monochromatic blue and red light influenced the photosynthetic and antioxidant system by inducing oxidative stress. The generation of excess ROS has resulted in the elicitation of antioxidant activity, which increased the contents of protective phytochemicals such as phenols and flavonoids (Figure 8). The proteomic analysis showed that blue and red light-mediated modulation of proteins is involved in photosynthesis, carbohydrate metabolism, and oxidative stress. Overall, the stress alleviation mechanism rendered the elicitation of secondary metabolites with medicinal value, and the red and blue lights also contributed to the physiological improvement of *S. kakudensis*.

## Conclusion

Application of novel approaches to enhance the micropropagation of indigenous medicinal plants has been increasing in recent days. In the present study, the impact of blue and/or red LEDs on the growth, accumulation of secondary metabolites, activities of antioxidant enzymes, and protein expression of *in vitro*-grown *S. kakudensis* has been demonstrated. The outcomes suggested that the blue or red LEDs can be utilized for the improvement of *in vitro* propagation of *S. kakudensis.* Moreover, the incorporation of red and blue LEDs elicited the synthesis of secondary metabolites in *S. kakudensis*. Taken together, the light qualities can be considered as an important abiotic elicitation cue for the production of secondary metabolites with therapeutic values. A better understanding of the molecular regulation of light quality-mediated changes at the protein level could facilitate the development of environmentally controlled regime for the production of medicinal plants with better physiological and phytochemical traits.

## Data Availability Statement

The original contributions generated for this study are included in the article/supplementary material, further inquiries can be directed to the corresponding author.

## Author Contributions

AM and BJ designed the experiments. AM and PS performed the experiments. AM wrote the manuscript. YP assisted in the SEM and phytochemical analysis. All authors have proofread and finalized the manuscript.

## Conflict of Interest

The authors declare that the research was conducted in the absence of any commercial or financial relationships that could be construed as a potential conflict of interest.

## Footnotes

1 http://www.matrixscience.com/search_form_select.html

2 http://www.ncbi.nlm.nih.gov/refseq
